# Surface Texturing of Polyethylene Terephthalate Induced by Excimer Laser in Silver Nanoparticle Colloids

**DOI:** 10.3390/ma14123263

**Published:** 2021-06-12

**Authors:** Jakub Siegel, Tatiana Savenkova, Jana Pryjmaková, Petr Slepička, Miroslav Šlouf, Václav Švorčík

**Affiliations:** 1 Department of Solid State Engineering, University of Chemistry and Technology Prague, 166 28 Prague, Czech Republic; savenkot@vscht.cz (T.S.); pryjmakj@vscht.cz (J.P.); slepickp@vscht.cz (P.S.); svorcikv@vscht.cz (V.Š.); 2 Institute of Macromolecular Chemistry, Academy of Sciences of the Czech Republic, Heyrovského nám. 2, 162 06 Prague, Czech Republic; slouf@imc.cas.cz

**Keywords:** polymer, silver nanoparticles, surface texturing, laser, surface morphology

## Abstract

We report on a novel technique of surface texturing of polyethylene terephthalate (PET) foil in the presence of silver nanoparticles (AgNPs). This approach provides a variable surface morphology of PET evenly decorated with AgNPs. Surface texturing occurred in silver nanoparticle colloids of different concentrations under the action of pulse excimer laser. Surface morphology of PET immobilized with AgNPs was observed by AFM and FEGSEM. Atomic concentration of silver was determined by XPS. A presented concentration-controlled procedure of surface texturing of PET in the presence of silver colloids leads to a highly nanoparticle-enriched polymer surface with a variable morphology and uniform nanoparticle distribution.

## 1. Introduction

Natural and synthetic polymeric materials are widely used in the manufacture of biomedical supplies [1,2,3]. Due to the versatility of their surface properties, which may easily be tailored by means of physical and chemical processes, polymers are suitable candidates for biomimetic structures in medical applications. These biomaterials are particularly used in tissue engineering [1], implants [4], drug-delivery carriers [5], wound healing materials or in biological imaging [6].

A common problem in current research of polymers for biomedical applications is the adhesion of bacteria and the formation of biofilms on the surface of medical devices. Bacteria cause infections and inflammation. To improve the bactericidal and anti-biofilm properties of biomaterials, various methods of their surface modification have been developed. The most common one is the coating of their surface with antibiotics. However, bacteria succeed in developing mechanisms to neutralize the effect of these therapeutics [7]. According to the World Health Organization, bacterial resistance to antibiotics, which is constantly increasing in all parts of the world, is today one of the greatest threats to health, agriculture and the food industry [8]. In addition, antibiotics have a number of disadvantages, including poor solubility, instability, and side effects. Therefore, alternatives to antibacterial therapy are intensively sought.

Antibacterial coatings of biocompatible polymers based on metal nanoparticles (NPs), especially gold, silver, and copper, have great potential in preventing or reducing the accumulation of biofilms on the surface of medical devices [9,10]. In particular, especially silver nanoparticles (AgNPs) are important candidates for the role of an alternative to conventional antibiotics. Their antimicrobial effect is associated with the release of silver Ag^+^ ions and the ability to interact with cell membranes [11]. Zerovalent AgNPs or inorganic silver compounds ionize in the presence of water, body fluids, or exudates. Formed silver ions are biologically active and can react with proteins, amino acid residues, as well as free anions and receptors located on eukaryotic cell membranes. The action of silver on bacteria and fungi is related to its passage through the cell wall and its ability to interact and irreversibly denature key enzyme systems [12]. Within the microorganism, silver generates free radicals that stop important metabolic processes in the cell, e.g., block electron transfer between respiratory chain enzymes, and enter into reactions with the thiol groups of oxidative enzymes. As a result of the disruption of vital functions, the cell gradually dies out [13].

In this work, we followed and deepened our previously published research in the field of laser-induced anchoring of silver NPs on polymers and demonstrated an effective way of tailoring the surface morphology of a polymeric carrier while its simultaneous enriching with AgNPs [14]. This research confirms that the formerly proposed mechanism of silver NP immobilization in polymers is correct and that a higher concentration of silver NPs in the immobilization solution has the same effect as decreasing the fluence of excitation radiation, due to an absorption effect in silver nanoparticle colloids of an increasing Ag concentration. This effect was demonstrated in a set of immobilization solutions, which varied in Ag concentrations ranging from 5 to 30 mg L^1^.

## 2. Materials and Methods

The PET samples (thickness 50 µm, supplied by Goodfellow Cambridge Ltd., Huntingdon, UK) were immersed in a colloid solution of silver nanoparticles (AgNPs) and irradiated by linearly polarized light from KrF excimer laser (fluence 18 mJ cm^−2^), according to the procedure published in [14].

The concentration of Ag in AgNPs colloids was determined by atomic absorption spectroscopy (Varian Inc., Palo Alto, CA, USA). Ultraviolet–visible spectroscopy (PerkinElmer Inc., Waltham, MA, USA) was used to study the optical properties of colloidal dispersions of AgNPs. AgNPs were visualized by transmission electron microscopy. Surface morphology and roughness was measured by atomic force microscope. Surface morphology of the samples was also analyzed by a high-resolution FEGSEM microscope (TESCAN, Brno, Czech Republic). Atomic concentration of Ag (Ag3d) on the surface of the samples was determined by X-ray photoelectron spectroscopy (Omicron Nanotechnology GmbH, Taunusstein, Germany). Instrumentation, measurement settings, detailed experimental conditions and measurement errors of individual analyzes are detailed in [14].

## 3. Results and Discussion

**Figure 1:**
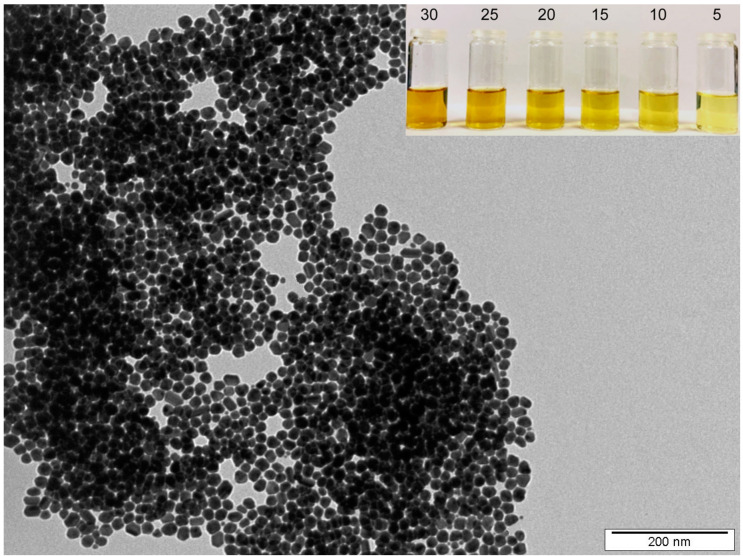
TEM image of as-synthesized silver nanoparticles. Inset shows AgNPs colloids with a decreasing Ag concentration (numbers stand for Ag concentration in mg L^−1^).

**Figure 2:**
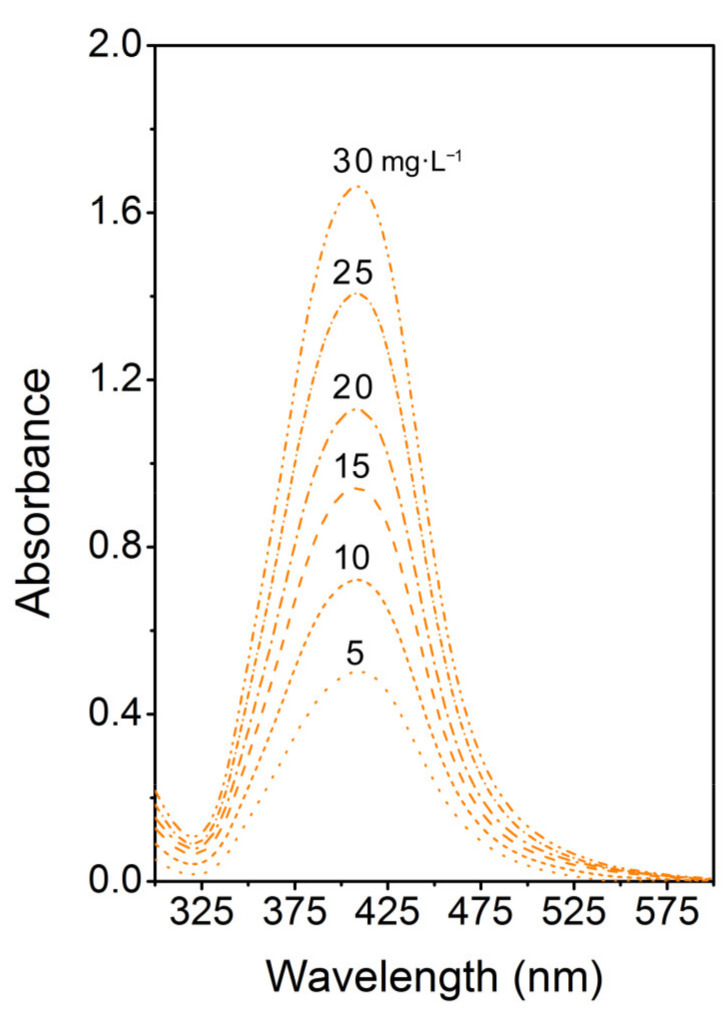
UV–Vis absorption spectra of AgNPs colloids of different Ag concentrations.

Figure 1 shows a typical TEM image of as-synthesized AgNPs colloids, the inset displays used AgNPs colloids of decreasing concentration (in mg L^−1^). Indeed, our synthesis procedure provides round-shape silver nanoparticles with a fairly narrow-size distribution. The concentration of Ag in as-synthesized AgNPs colloids was 45 mg L^−1^. As-synthesized AgNPs were diluted by buffer solution (0.4 mM sodium citrate) for irradiation purposes with 5, 10, 15, 20, 25, and 30 mg L^−1^. After diluting the solutions to the desired concentrations, UV–Vis analysis was performed (Figure 2) to verify the colloid stability. UV–Vis spectroscopy is often used to estimate the size, shape, and particle distribution of colloid solutions of metal nanoparticles since they show a specific absorption band corresponding to localized surface plasmon resonance (LSPR) [15]. Regarding the composition of AgNPs solution (0.4 mM of sodium citrate in water), the specific shape of the absorption bands truly corresponds to round-shaped NPs of narrow-size distribution and a mean diameter around 25 nm [16], which corresponds well with the TEM analysis. The position of the LSPR maxima at 409 nm remains practically unchanged in the case of all examined solutions which points to particle aggregation resistance and a good colloid stability. A recorded increase in intensity corresponds to the increasing concentration in AgNPs.

**Figure 3:**
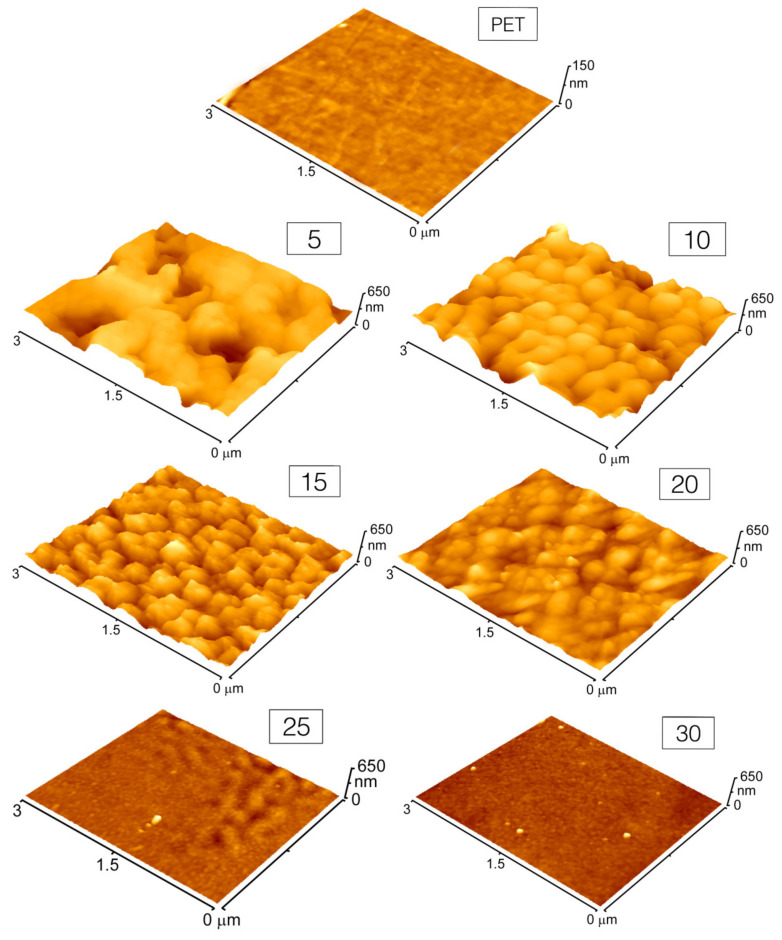
AFM images of PET after its irradiation in the presence of AgNPs colloids of different Ag concentrations. Numbers in the boxes indicate the concentration of silver in the immobilization solution. (Notice: Z-axis scaling in the case of pristine PET is limited to 150 nm due to naturally flat surface morphology).

Immediately after PET irradiation in AgNPs colloids, the samples were rinsed by distilled water, dried with a nitrogen stream, and placed into a desiccator for 24 h. Surface morphology of pristine and AgNPs immobilized PET was studied by AFM and is depicted in Figure 3. 

**Figure 4:**
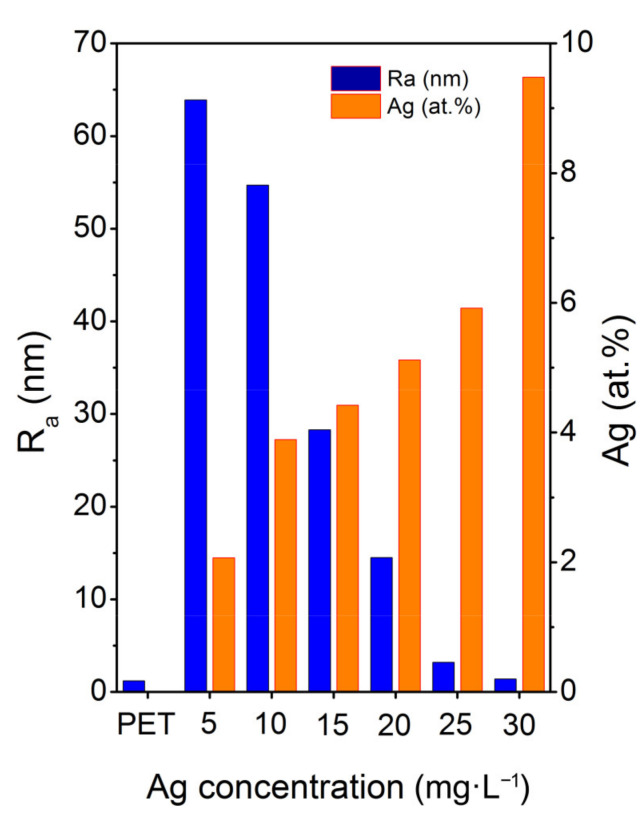
Average surface roughness (Ra) and atomic concentration of silver determined by XPS as a function of the Ag concentration in the immobilization solution.

Pristine PET exhibits a flat surface with surface roughness Ra about 1 nm (see Figure 4, blue bar), which is typical for commercially available foils. Once PET is laser irradiated in the presence of AgNPs colloids, the surface morphology changes dramatically, corresponding to AgNPs incorporation to the polymer surface and transformation of the surface morphology due to light absorption. The development of the surface morphology of the polymer is in accordance with the immobilization mechanism of NPs by the action of intense light of wavelengths near the maximum of their plasmon resonance, described in detail in our previous work [14]. As the Ag concentration in the immobilization solution increases, a proportional dose of light energy is absorbed due to plasmon excitation in AgNPs, which reduces the part of the energy absorbed by the polymer surface and, thus, prevents a change in its surface morphology. Thus, PET immobilized at AgNPs colloids of higher concentration (30 mg L^−1^) contains the highest amount of silver (see Figure 4, orange bar) while its surface morphology is quite flat, reminding that of a pristine polymer. On the other hand, when the immobilization solution contains the lowest concentration of AgNPs (5 mg L^−1^), surface roughness increases significantly (see Figure 4, blue bar) and the detected amount of Ag drops down (Figure 4, orange bar).

**Figure 5:**
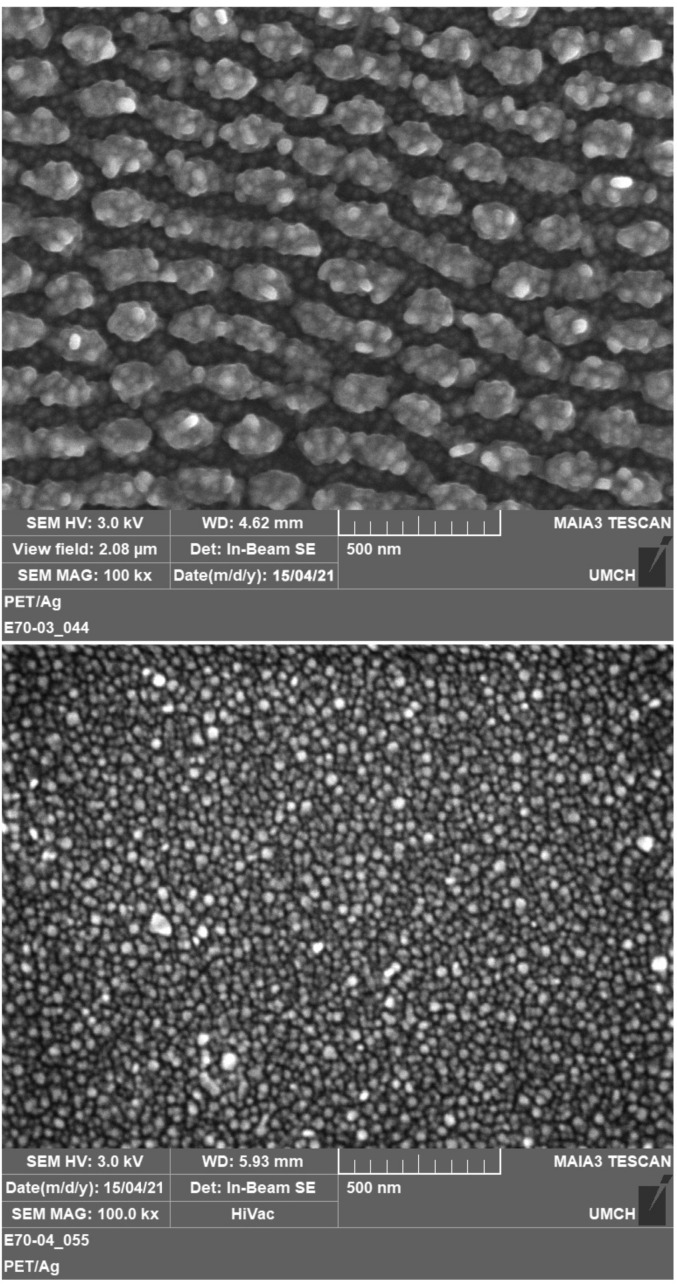
FEGSEM micrographs showing the surface morphology of PET after its irradiation in the presence of AgNPs colloids of different Ag concentrations: 15 mg L^−1^ (up) and 30 mg L^−1^ (down).

We completed an AFM observation with FEGSEM analysis (Figure 5) to better visualize AgNPs on the surface of PET. Figure 5 shows corrugated and smooth PET foils with Ag nanoparticles immobilized in colloids with a Ag concentration of 15 and 30 mg L^−1^, respectively. Presented micrographs illustrate that the morphology is quite uniform, with evenly distributed AgNPs over the polymer surface. It is evident that the irradiation in higher concentrated AgNPs solution resulted in a smooth surface with planar morphology decorated with nanoparticles, while under lower concentration a rough and rugged polymer surface was obtained. Those findings are in good accordance with AFM. They also point to the key issue of concentration-controlled immobilization compared to the laser fluence-controlled approach, presented in our former study [14]. The concentration-controlled approach benefits from the application of constant laser fluence, which strictly preserves its effect on particle size through the immobilization process. Thus, the immobilized particles under the concentration-controlled approach are of the same size regardless of the resulting polymer morphology (smooth or rough). This does not apply in the case of the fluence-controlled approaches, where an increase in laser fluence (leading to a rough surface morphology) causes an increase in particle size due to considerably higher absorbed energy and promoted particle ingrowth [17].

It is also worth emphasizing that, as the Ag concentration in the immobilization solution increases, the silver concentration over the PET surface increases proportionally (see Figure 4), while the *R*_a_ value monotonously decreases as the surface morphology becomes smoother owing to the fact that more incoming energy is absorbed by the particles.

## 4. Conclusions

In this work we introduced a simple and versatile approach for laser induced texturing of PET foil in the presence of silver nanoparticles. This method is based on laser processing of polymers in the presence of AgNPs colloids of variable concentration. Compared to laser fluence-controlled approaches, our procedure enables to maintain a size of nanoparticles while controlling the resulting morphology of the polymer. We demonstrated that within the AgNPs concentration range of 5–30 mg L^−1^, where a rough and smooth polymer morphology may be produced, the PET foil was evenly decorated by silver nanoparticles. An increasing concentration of silver in the immobilization solution leads to a monotonic increase in the concentration of silver detected on the polymer surface. Surface texturing of polymers highly enriched with silver nanoparticles can create new opportunities in the development of prospective antimicrobial coatings, especially suitable for inhibition of biofilm formation.

## Data Availability

The data presented in this study are available on request from the corresponding author.
